# Structural Basis of the Light‐Switchable Interaction between an Azobenzene Side Chain in a Biosynthetic Protein and *α*‐Cyclodextrin

**DOI:** 10.1002/open.202500471

**Published:** 2025-11-19

**Authors:** Andreas Eichinger, Peter Mayrhofer, Markus R. Anneser, Leonie Jarzinka, Arne Skerra

**Affiliations:** ^1^ Chair of Biological Chemistry School of Life Sciences Technical University of Munich Freising Germany

**Keywords:** azobenzene, *cis*/*trans* isomerization, E/Z isomerization, excitography, noncanonical amino acid

## Abstract

Azobenzene derivatives, which show light‐induced reversible *trans*↔*cis* isomerization, have gained increasing attention in the area of protein science. *p*‐(Phenylazo)‐L‐phenylalanine (Pap) was recently employed to enable the light‐controlled affinity purification of biosynthetic proteins as part of the Azo‐tag. Specific supramolecular complex formation with immobilized *α*‐cyclodextrin (*α*‐CD) groups is mediated by the Pap side chain in its low‐energy *trans*‐configuration, whereas photoisomerization to the *cis*‐state leads to immediate dissociation. Here, we describe the X‐ray crystallographic analysis of super‐folder green fluorescent protein (sfGFP) displaying Pap at amino acid position 39 on its surface in complex with *α*‐CD. While this experimental structure generally confirms the mode of host–guest interaction predicted by molecular modeling, there are two unexpected observations: (i) the conically shaped *α*‐CD binds with its narrow end toward the aminoacyl moiety of Pap, despite appearing sterically more demanding, and (ii) the azobenzene side chain shows a considerably twisted conformation of its two phenyl rings, which contrasts with the fully coplanar arrangement usually anticipated for unmodified azobenzene and its chemical derivatives. Thus, this crystal structure of the photoswitchable noncanonical amino acid Pap (also known as AzoF or AzoPhe) provides valuable insight for future molecular engineering endeavors to endow proteins with light‐controllable functions.

## Graphical abstract

X‐ray crystallographic analysis of super‐folder green fluorescent protein (sfGFP), a common reporter protein, displaying *p*‐(phenylazo)‐L‐phenylalanine (Pap) at amino acid position 39 on its surface in complex with *α*‐cyclodextrin (*α*‐CD) reveals an unexpected conformation of the photoswitchable side chain and illustrates its light‐dependent supramolecular complex formation, which can be applied for affinity purification.

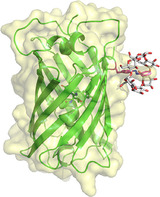

## 1 Introduction

The recent development of light‐controlled affinity chromatography, dubbed Excitography, has opened a new era in the purification of recombinant proteins and, potentially, other biomolecules [1]. In this technique, the interaction between the protein of interest (POI) and the affinity matrix is governed by a derivative of azobenzene, which can reversibly change its chemical configuration (*trans*↔*cis*) if illuminated with light at a specific wavelength, thus either allowing adsorption to the stationary phase or triggering dissociation and elution with the mobile phase. Notably, in this kind of chromatography, the application of special chemical or buffer conditions, such as low/high pH, elevated salt concentrations, denaturants, chelating agents, or competing ligands, for elution is not required since adsorption/elution is solely controlled by the effect of the electromagnetic radiation. Hence, the POI can be conveniently obtained in a buffer of choice, directly suitable for subsequent biochemical or biological experiments.

**SCHEME 1:**
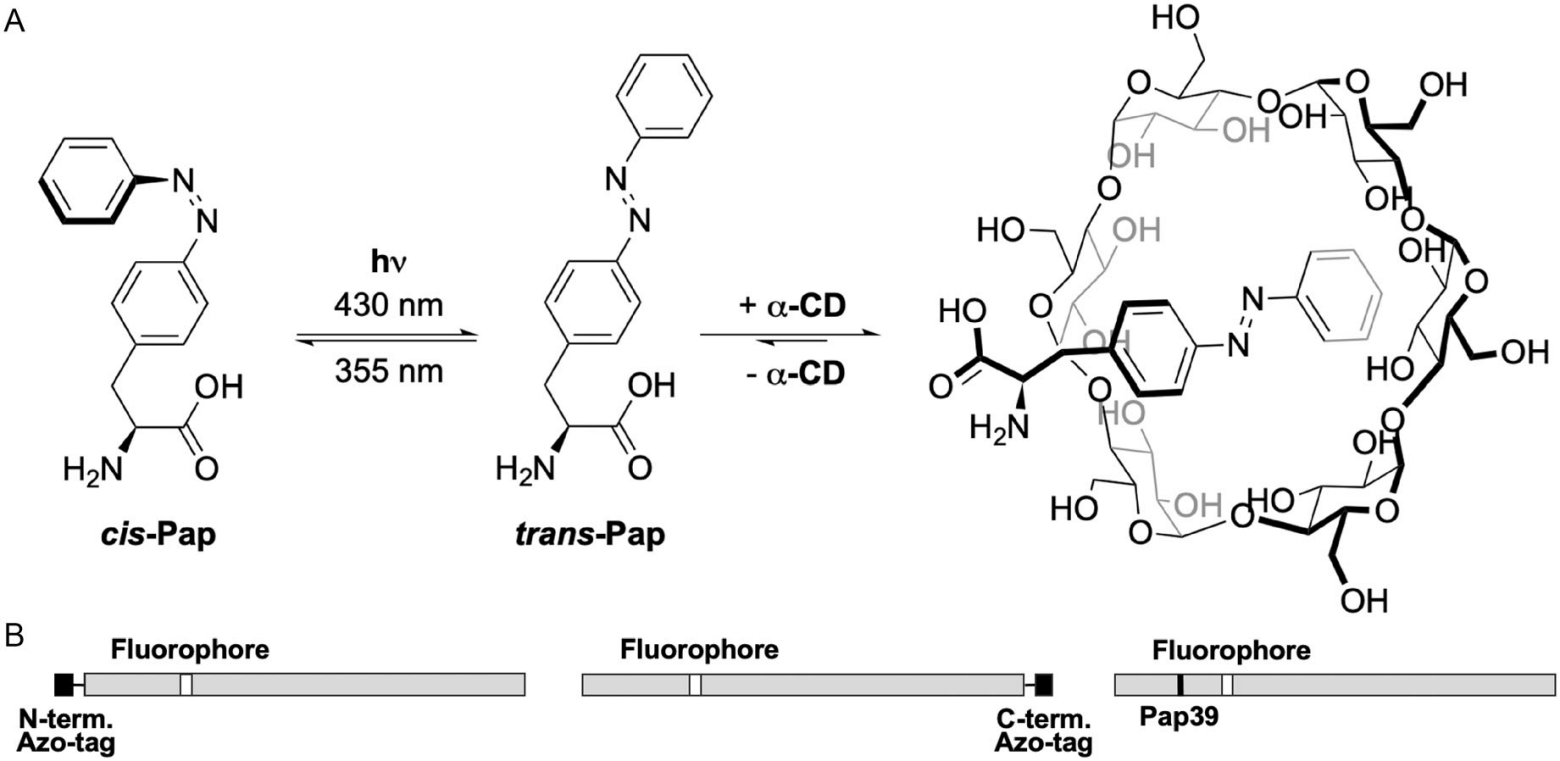
Primary structure and light–dependent complex formation of sfGFP^39Pap^ with *α*‐CD. (A) Principle of the light‐controlled host/guest complex formation between α‐CD and the Pap side chain exclusively in its *trans*‐configuration (i.e., the energetically favored ground state) [1]. (B) Bar diagram: sfGFP with the Azo‐tag at the N‐terminus, at the C‐terminus, or Pap incorporated at position 39 (see Supporting Information Figure S1).

The first implementation of this novel approach in protein purification was based on the Azo‐tag, comprising the noncanonical amino acid *p*‐(phenylazo)‐L‐phenylalanine (Pap), whose side chain can be quantitatively switched from its *trans* ground state to the metastable *cis*‐configuration by irradiation with mild UV‐A light at 355 nm wavelength. Pap can be efficiently incorporated into recombinant proteins by way of an expanded genetic code utilizing amber stop codon suppression [1]. Surrounded by small adjacent amino acid residues like Gly, Ala, or Pro, the Pap side chain in its *trans*‐configuration forms a stable complex with *α*‐cyclodextrin (*α*‐CD). Thus, if an aqueous solution of the Azo‐tagged POI is applied to a chromatography column packed with a hydrophilic translucent matrix that carries covalently immobilized *α*‐CD groups, the POI adsorbs to this matrix in a highly specific manner. Other macromolecules in the solution, in particular host cell proteins from the genetically modified production organism, are quickly washed out with the buffer flow. However, as soon as the column with the bound Azo‐tagged POI is illuminated with UV‐A light, the Pap side chain switches to the bulky *cis*‐configuration, which cannot form a host/guest complex with *α*‐CD (Scheme 1A), and this leads to the quick elution from the column. In this manner, the POI is obtained in a highly pure state in a mild buffer of choice, as demonstrated for a broad range of biochemically or biologically relevant proteins, from fluorescent proteins to antibodies and their fragments [1, 2].

## 2 Experimental Section

### 2.1 Preparation of the sfGFP^39^ ^Pap^ Protein and Light‐Controlled Affinity Chromatography

sfGFP^39Pap^ was expressed from the pSB19 plasmid encoding the reading frame for the full‐length protein with the C‐terminal *Strep*‐tag II as well as an amber stop codon at amino acid position 39 (Supporting Information Figure S1) and purified via *α*‐CD affinity chromatography as described for Azo‐tagged POIs [1]. Briefly, the engineered *E. coli* strain NEBExpress(lowRF1) transformed with pSB19‐PapRS#34‐sfGFPa39 was grown at 30°C in 100 ml LB/Amp medium to a cell density OD_550_ ≈ 0.4. Then, 0.2 mM Pap, 0.8 mM 2‐hydroxypropyl‐*β*‐CD and 0.2% (w/v) arabinose were added (from appropriate stock solutions), followed by the addition of 0.5 mM isopropyl‐*β*‐D‐thiogalactopyranoside (IPTG) at OD_550_ ≈ 0.8, for induction of recombinant gene expression and biosynthetic incorporation of the noncanonical amino acid. After further incubation for 18 h at 30°C, the bacteria were harvested by centrifugation and lysed via sonication using a suspension in 4 ml chromatography buffer (25 mM Tris/HCl, 150 mM NaCl, pH 8.0).

**FIGURE 1:**
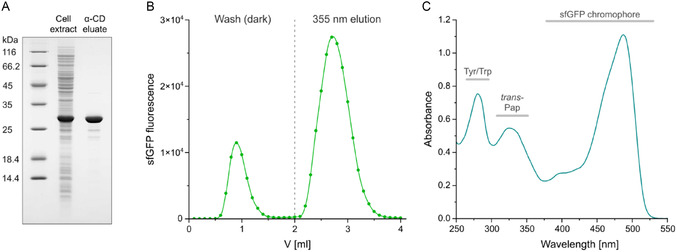
Purification of sfGFP^39Pap^ via light‐controlled *α*‐CD affinity chromatography. (A) A cleared lysate prepared from *E. coli* NEBExpress(lowRF1) cells expressing sfGFP^39Pap^ (“Cell extract”) was applied to a 1 mL *α*‐CD affinity column, and host cell constituents were washed out with buffer at physiological pH. sfGFP^39Pap^ was eluted in a highly pure state (“*α*‐CD eluate”) upon illumination with 355 nm UV‐A light by effecting *trans*→*cis* isomerization of the Pap side chain. (B) After reverting Pap predominantly to its energetically favored *trans*‐state by exposure to daylight, 100 µl (0.3 mg sfGFP^39Pap^) of the eluate from (A) was again applied to a 1 mL *α*‐CD affinity column. Washing and elution were monitored by the sfGFP fluorescence in 100 µl fractions. 20% of the total fluorescence was found in the wash fractions, which corresponds to the small proportion of Pap residues in the *cis*‐configuration according to the photostationary state (PSS) under daylight [1], whereas the major proportion of sfGFP with Pap in the *trans*‐state was retained on the column until exposure to UV‐A light. (C) Absorption spectrum of the purified sfGFP^39Pap^, which exhibits an additional peak around 330 nm wavelength characteristic for the *trans*‐Pap residue.

To demonstrate the light‐controlled affinity purification of sfGFP^39Pap^, 2 ml of the cleared lysate was applied to an *α*‐CD affinity column with 1 ml bed volume, followed by washing with 3 ml chromatography buffer in a shaded laboratory. sfGFP^39Pap^ was then eluted in 1.5 ml chromatography buffer via illumination with LEDs emitting 355 nm UV‐A light. The eluate was exposed to daylight for 1 h and, to quantitatively investigate binding of the biosynthetic protein to the *α*‐CD affinity column (see Figure 1), 100 µl containing ≈0.3 mg of the purified sfGFP^39Pap^ was again applied. The *α*‐CD affinity column was washed with 2 ml chromatography buffer in the dark, followed by 2 ml of the same buffer under 355 nm illumination. During washing and elution, 100 µl fractions were collected and the sfGFP fluorescence was quantified with a Synergy 2 plate reader (BioTek, Winooski, VT) using 485/20 and 528/20 nm filters for excitation and emission, respectively.

For the purpose of protein crystallization, sfGFP^39Pap^ was produced in 2 l LB/Amp medium at 30°C, followed by the addition of Pap and arabinose at OD_550_ ≈ 0.4 and induction of gene expression with IPTG at OD_550_ ≈ 0.8, as above. Bacteria were harvested by centrifugation and resuspended in 20 ml 100 mM Tris/HCl, 150 mM NaCl, 1 mM EDTA, pH 8.0, followed by disruption in a French pressure cell (SLM Aminco, Urbana, IL). sfGFP^39Pap^ was purified via its C‐terminal *Strep*‐tag II on a StrepTactin affinity column using elution with D‐desthiobiotin. The eluted protein was dialyzed against 20 mM Tris/HCl, pH 8.0 and subjected to anion exchange chromatography (AEX) on a Resource Q column (Cytiva, Munich, Germany), followed by elution with a 0–500 mM NaCl concentration gradient in the same buffer, yielding in total 25–30 mg pure monomeric protein. The resulting protein was dialyzed against 20 mM Tris/HCl, pH 7.5, concentrated in a 15 ml Amicon Ultra Centrifugal Filter, 10 kDa MWCO (Merck Millipore, Darmstadt, Germany), and sterile filtered using Ultrafree Centrifugal Filters PVDF 0.22 μm (Merck Millipore). Protein purification was checked by SDS‐PAGE (Supporting Information Figure S2) and ESI‐MS on an impact II mass spectrometer (Bruker Daltonics, Bremen, Germany).

### 2.2 Protein Crystallization and X‐ray Data Collection

To cocrystallize sfGFP^39Pap^ with *α*‐CD, a 100 mM stock solution of *α*‐CD (Sigma–Aldrich, Taufkirchen, Germany) in 20 mM Tris/HCl, pH 7.5 (sterile‐filtered) was prepared and added at a 1:10 volume ratio to the solution of 48 mg/ml purified sfGFP^39Pap^ from above.

A search for suitable precipitation conditions was performed using an in‐house precipitant screen with sitting drops consisting of 600 nl protein and 200 nl precipitant solution. A single crystal was obtained at 20°C after ≈75 days in the dark with 3 M NaCl, 0.1 M Na‐acetate, pH 4.5 as precipitant. The crystal was transferred into the precipitant buffer supplemented with 25% (v/v) glycerol as cryoprotectant and immediately frozen in liquid nitrogen. A synchrotron X‐ray diffraction data set was collected under cryo‐conditions at beamline P14 of the PETRA III storage ring operated by EMBL (DESY, Hamburg, Germany). The complex crystallized in the space group P4_3_ (Supporting Information Table S1), with two protein complexes in the asymmetric unit (a.u.).

### 2.3 Diffraction Data Processing, Crystallographic Analysis, and Structural Refinement

X‐ray diffraction data were processed and scaled with the XDS software package [3]. Molecular replacement was performed with Phaser from the CCP4 program suite [4] using the coordinates of sfGFP (PDB ID: 2B3P) as search model. The molecular model of *α*‐CD (PDB ID: 5YNA) was adopted from a cocrystal structure with Pullulanase from *Klebsiella pneumoniae* [5]. The atomic model of the complex was built with Coot [6] and refined with Refmac5 [7] using topology files for Pap and *α*‐cyclodextrin generated with PRODRG [8] (from the CCP4 program suite).

Water molecules were added using ARP/wARP (from CCP4). The structural model was rectified using the PDB‐REDO server [9] and finally validated with the MolProbity server [10]. Secondary structures were assigned using DSSP [11]. Protein‐ligand contact surfaces were calculated with PISA [12], and molecular graphics were prepared using PyMOL software (Schrödinger, New York, NY). The angle (*α*) between the two phenyl ring planes in Pap (or other azobenzene derivatives) was calculated with PyMOL ver. 3.1.6.1 using a Python script (Supporting Information Figure S3).

To parametrize the topology file for the Pap residue, in total four atomic planes were defined as constraints: one for the main chain atoms of the peptide group (C*α*, C, and O), each one for the two aromatic rings and one for the azo group in between (together with the directly connected carbon atoms). When the structural model of sfGFP^39Pap^ was alternatively refined with a topology file wherein the entire azobenzene side chain was constrained onto one single plane, the *R*_cryst_ and *R*_free_ factors were slightly elevated and the resulting F_o_–F_c_ difference electron density clearly indicated a deviating twisted conformation of the outmost phenyl ring. On the other hand, the omission of the constraint for the azo group in the initial topology file (which was used for the final refinement) did not influence the side chain conformation of Pap, its electron density, or the *R*_cryst_ and *R*_free_ factors.

The fully refined atomic model of sfGFP^39Pap^ in complex with α‐CD revealed electron density for 448 of in total 494 amino acid residues present in the a.u.: Ser2–Gly228 of chain A as well as Lys3–Tyr74 and Lys79–Ala227 of chain B. Chain A was selected for further structural analysis based on the better quality of its electron density map.

## 3 Results and Discussion

To elucidate the structural basis of the noncovalent complex formation between the Pap side chain in its energetically favored *trans*‐configuration and the *α*‐CD group, we have crystallized super‐folder green fluorescent protein (sfGFP), serving as a well‐characterized model POI exposing the Pap side chain on its surface, in the presence of *α*‐CD and performed X‐ray crystallographic analysis at 2.05 Å resolution. To minimize flexible extensions of the polypeptide chain, which are known to hamper crystal packing and often assume disordered conformations lacking defined electron density, we did not employ an N‐ or C‐terminal Azo‐tag. Instead, we introduced the Pap residue at the structurally permissible position of Tyr39 (mutated to Asn in sfGFP) on the surface of the folded protein (Scheme 1B). The same position was utilized in a preceding study [13] for the incorporation of a fluorescent hydroxycoumaryl side chain into enhanced cyan fluorescent protein (eCFP). Notably, in spite of the sterically more restricted situation on the surface of the core protein, compared with the flexible Azo‐tag [1], sfGFP^39Pap^ was successfully recovered by light‐controlled affinity purification from the total cell extract of *E. coli* (Figure 1).

**FIGURE 2:**
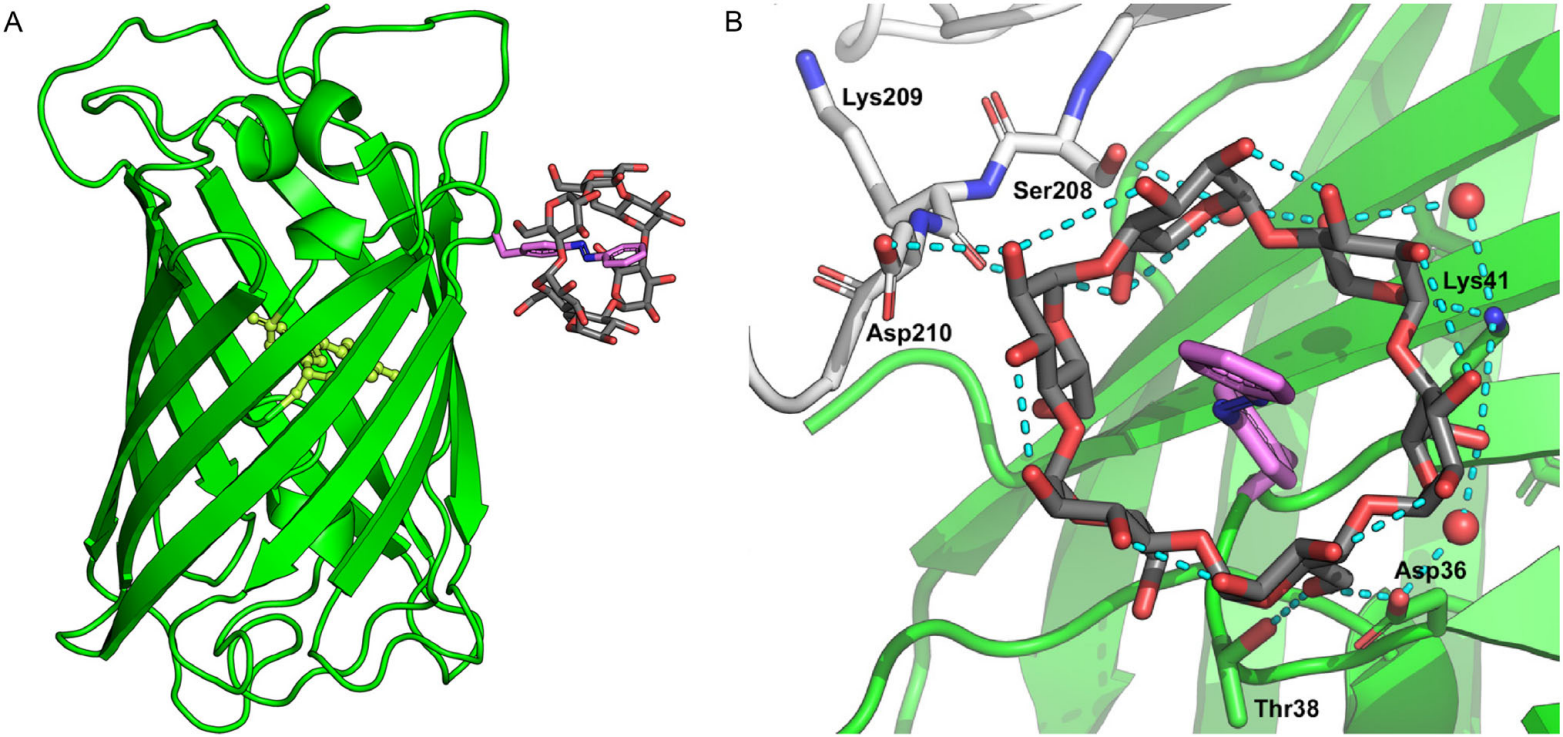
Crystal structure of sfGFP^39Pap^ in complex with *α*‐CD. (A) Overview: sfGFP shown as ribbon diagram, with the fluorophore as spheres and the Pap side chain, as well as *α*‐CD, depicted as sticks. (B) Close‐up view of the host/guest complex between the Pap side chain and *α*‐CD, including its network of hydrogen bonds (contributing protein residues are labeled).

The recombinant protein (Supporting Information Figure S2) was crystallized in the dark in the presence of 10 mM *α*‐CD (and 3 M NaCl, 0.1 M Na‐acetate, pH 4.5 as precipitant), and its X‐ray analysis revealed two sfGFP^39Pap^ molecules per asymmetric unit (a.u.). As anticipated, both molecules revealed the characteristic ring‐like electron density of *α*‐CD surrounding the azobenzene side chain in its *trans*‐state (Figure 2). While monomer A shows a generally better defined electron density, both complexes within the a.u. share high structural similarity, thus indicating that the association of each Pap side chain with *α*‐CD is not significantly influenced by the crystal packing environment. Furthermore, the fold of each sfGFP molecule is strongly conserved compared with the crystal structure of the original protein [14], including a fully matured fluorophore, with root mean square deviations (RMSDs) of 0.775 between molecules A and B (C*α* positions 3–74 and 79–227) and of 1.306 Å between molecule A and the unmodified sfGFP (C*α* positions 2–228).

**FIGURE 3:**
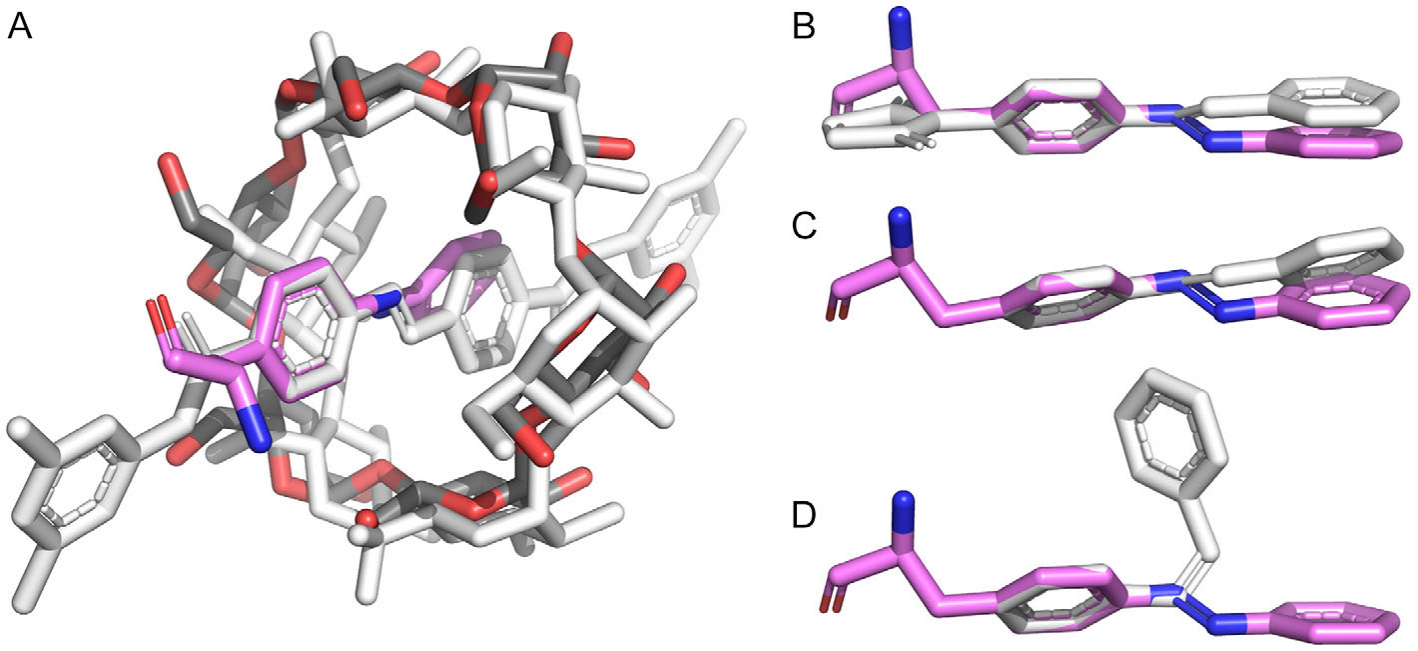
The geometry of the azobenzene side chain in the noncanonical L‐amino acid Pap compared with azobenzene derivatives from the CSD (see Table 1). Superposition between the Pap residue as part of the sfGFP^39Pap^•*α*‐CD complex (colored) and (A) an *α*‐CD•azobenzene rotaxane complex (light gray), (B) maleimidyl‐azobenzene, (C) *trans*‐azobenzene, and (D) *cis*‐azobenzene. In each case, the C‐atoms of the first phenyl ring and the first N‐atom of the azo group were used for the superposition.

The mode of host/guest complex formation between the Pap side chain at position 39 of sfGFP and *α*‐CD exhibits several remarkable features. First, the azobenzene moiety is almost centrally buried within the hydrophobic interior of *α*‐CD, with the azo group positioned close to the narrowest diameter of the sugar ring. This not fully symmetric mutual arrangement is apparently not caused by sterical hindrance imposed by the peptide backbone or by the sfGFP surface from which the Pap side chain protrudes; in fact, superposition with a small‐molecule complex between *α*‐CD and an azobenzene as part of a rotaxane [15], which is sterically locked with bulky substituents at both *para*‐positions of the azobenzene, indicates strong structural similarity (Figure 3). By this mode of interaction, the accessible surface area of the Pap side chain is reduced from 273.4 Å^2^ in sfGFP^39Pap^ alone to 65.5 Å^2^ in the complex with *α*‐CD (both compared to a much larger surface of 467.3 Å^2^ for the isolated Pap amino acid, calculated for the same conformation as seen in sfGFP^39Pap^).

The *α*‐CD ring structure, which is made of six D‐glucose residues, shows a slightly distorted toroidal shape that is often described as a conus with a wider and a more narrow opening [16, 17]. Interestingly, in the complex with the Pap side chain, *α*‐CD points toward the sfGFP surface with its narrow opening. At first glance, this could represent a sterically more tense situation; however, at closer inspection, it becomes evident that this mode of complex formation is stabilized by a circular network of hydrogen bonds between the sugar hydroxyl groups and several side chains of sfGFP^39Pap^ that surround the Pap residue, including the preceding Thr side chain (Figure 2B), as well as some residues from the neighboring sfGFP^39Pap^ molecule within the a.u. On the other hand, the C2‐OH and C3‐OH groups on the opposite side (with the wider opening) of the *α*‐CD conus are mostly engaged in intramolecular hydrogen bonds that bridge adjacent D‐glucose units and form a polar interface with the aqueous solvent.

**FIGURE 4:**
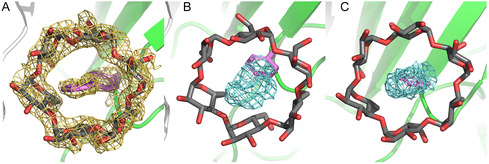
Electron density around the sfGFP^39Pap^•*α*‐CD complex and geometry of the Pap side chain. (A) 2F_o_–F_c_ electron density contoured at 1 *σ* (light orange) for Pap and *α*‐CD. (B) Omit map for the Pap side chain calculated by refinement of the protein structure using a Phe residue at position 39 instead. The well‐defined positive F_o_–F_c_ difference density contoured at 1 *σ* (cyan) clearly reveals the presence of the azo group and of the second phenyl ring. (C) View of the same positive F_o_–F_c_ difference electron density along the aromatic plane of the Phe side chain, demonstrating that the outer phenyl ring (of Pap) is tilted with regard to the inner phenyl ring counter‐clockwise by >30°.

Furthermore, the conformation of the Pap side chain itself deserves attention. The classical crystal structure of plain *trans*‐azobenzene indicated a coplanar arrangement of both aromatic phenyl rings, including the *π*‐conjugated azo group in between [18]. This rigid, fully planar conformation has been anticipated for molecular models of azobenzene and its derivatives in general [19]. In contrast, however, the Pap side chain in the present crystal structure considerably deviates from this presumed ideal stereochemistry, with an angle of 35.5° between the inner and the outer aromatic ring planes (Table 1 and Figure 4).

**TABLE 1:** Dihedral angles around the N=N group (*χ*) and mutual angle between the two phenyl ring planes (*α*) for azobenzene and relevant derivatives, including Pap (as incorporated in sfGFP^39Pap^).

Compound	Database ID	Ref.	***χ***_**pre**_	***χ***_**azo**_	***χ***_**post**_	***α***_**aromates**_
*trans*‐Azobenzene	1 104 320 (CSD)	[18]	−18.2°	180.0°	18.2°	0.0°
*p*,*p*′‐Azotoluene	1 104 392 (CSD)	[20]	−11.2°	180.0°	11.2°	0.0°
*α*‐CD•bis(*p*‐((3,5‐dimethylphenyl)amino)carbonyl)azobenzene rotaxane	675 081 (CSD)	[15]	2.8°	177.9°	−1.9°	4.8°
Bis(*p*‐((3,5‐dimethylphenyl)amino)carbonyl)azobenzene	675 083 (CSD)	[15]	−11.7°	180.0°	11.7°	0.0°
*cis*‐Azobenzene	1 104 150 (CSD)	[21]	−57.0°	−7.7°	−57.0°	115.7°
Maleimidyl‐azobenzene	845 384 (CSD)	[22]	21.5°	−177.1°	24.3°	45.9°
*Gluazo*	4H8I (PDB)	[23]	−0.3°	−179.8°	0.3°	179.7
sfGFP^39Pap^•*α*‐CD	9S0T (PDB)	this study	62.9°	−179.1°	−28.2°	35.5°

This unexpected observation prompted us to have a closer look at the other three‐dimensional structures of azobenzenes (Table 1), whose coordinates are accessible at the Cambridge Structural Database (CSD) or the Protein Data Bank (PDB). Structural comparison revealed that, like the prototypic azobenzene (Figure 3C), the closely related compound *p*,*p*′‐azotoluene and also the azobenzene moiety from the bis(*p*‐(((3,5‐dimethylphenyl)amino)carbonyl)azobenzene rotaxane (the latter both in complex with *α*‐CD and in the free state; cf. Table 1) show an extended fully planar structure (Figure 3A). In contrast, the monosubstituted maleimidyl‐azobenzene exhibits a tilted appearance very similar to the one seen for Pap in the sfGFP^39Pap^ crystal structure (Figure 3B), with an even larger angle of 45.9° between the two phenyl ring planes (albeit smaller distortion of the dihedral angle between the first phenyl ring and the N=N group). In contrast, the structure of azobenzene in the *cis*‐state is clearly different (Figure 3D).

Hence, it appears that asymmetrically or mono‐substituted azobenzene derivatives, such as the noncanonical L‐amino acid Pap, in the energetically favored *trans*‐state tend to adopt a nonplanar arrangement of the two aromatic rings connected by the planar *trans*‐azo group. Inevitably, this should lead to a weakening of the conjugation between the two corresponding Hückel *π*‐electron ring systems, which might deserve further investigation by quantum‐mechanical calculations. Nevertheless, the structural situation for substituted azobenzenes in general seems to be less clear since, for example, the derivative (4R)‐4‐[(2E)‐3‐{4‐[(E)‐phenyldiazenyl]phenyl}prop‐2‐en‐1‐yl]‐L‐glutamic acid, a synthetic photochromic ligand dubbed *gluazo* (see Table 1), was observed in a fully planar configuration when bound to the kainate receptor [23].

## 4 Conclusions

Our X‐ray crystallographic study of an sfGFP mutant exhibiting the Pap side chain in complex with *α*‐CD confirms the previously postulated mechanism of host/guest interaction between this small cyclodextrin and, specifically, azobenzene in its lower‐energy *trans*‐configuration which provides the basis for the light‐controlled affinity chromatography of Azo‐tagged proteins, termed Excitography. Unexpectedly, we found that the *trans*‐azobenzene side chain does not show the fully planar configuration as generally assumed in the scientific literature, but adopts a significantly twisted shape. This is an important structural feature to guide future protein engineering endeavors when implementing the Pap residue into proteins in order to control their functional properties via light‐induced *trans*↔*cis* isomerization.

## Supporting Information

Additional supporting information can be found online in the Supporting Information section. **Supporting Fig.** **S1:** Amino acid sequence of sfGFP^39Pap^ aligned against the one of wild‐type GFP from *Aequorea victoria* (UniProt‐ID: P42212) and with secondary structure assignments according to the present X‐ray structural analysis. Differing residues are highlighted and the Pap residue (X) at position 39 is indicated by a star. **Supporting Fig.** **S2** **:** ESI mass spectrum of the purified sfGFP^39Pap^ that was used for cocrystallization with α‐CD. Inset: Coomassie‐stained SDS‐PAGE of the purified sfGFP^39Pap^. Calculated mass for the protein with processed N‐terminal Met residue and mature fluorophore: 27,966.45 Da. The small peak at higher mass corresponds to a minor contamination by the recombinant protein that has the start Met residue retained (calc.: 28,097.57 Da). **Supporting Fig.** **S3** **:** Python script “AromaticAngle.py” to calculate the angle (α) between the two phenyl ring planes in Pap (or other azobenzene derivatives) to be executed using PyMOL ver. 3.1.6.1 software (adapted from https://pymol‐users.narkive.com/O0BZdPOe/pymol‐how‐to‐measure‐the‐angle‐between-two‐aromatic‐rings). **Supporting Table** **S1:** X‐ray data statistics.

## Funding

This work was supported by Deutsches Elektronen‐Synchrotron DESY (2025‐MX‐1019), and Deutsche Forschungsgemeinschaft (INST 95/1734‐1).

## Conflicts of Interest

The authors declare no conflicts of interest.

## Supporting information

Supplementary Material

## Data Availability

The data that support the findings of this study are available in the supplementary material of this article and from the Protein Data Bank (PDB) at https://www.rcsb.org under accession code 9S0T.

## References

[1] P. Mayrhofer , M. R. Anneser , K. Schira , et al., Nature Communications 15 (2024): 10693. 10.1038/s41467-024-55212-y PMC11655525 39695158

[2] F. Veitl , A. Eichinger , P. Mayrhofer , et al., ChemBioChem 26 (2025): e2500102. 10.1002/cbic.202500102 PMC12135134 40085144

[3] W. Kabsch , Acta Crystallographica Section D Structural Biology 66 (2010): 125–132. 10.1107/S0907444909047337 PMC2815665 20124692

[4] A. J. McCoy , R. W. Grosse‐Kunstleve , P. D. Adams , M. D. Winn , L. C. Storoni , and R. J. Read , Journal of Applied Crystallography 40 (2007): 658–674. 19461840 10.1107/S0021889807021206 PMC2483472

[5] N. Saka , H. Iwamoto , D. Malle , N. Takahashi , K. Mizutani , and B. Mikami , Acta Crystallographica Section D, Structural Biology 74 (2018): 1115–1123. 30387770 10.1107/S2059798318014523

[6] P. Emsley , B. Lohkamp , W. G. Scott , and K. Cowtan , Acta Crystallographica Section D, Structural Biology 66 (2010): 486–501. 10.1107/S0907444910007493 PMC2852313 20383002

[7] G. N. Murshudov , A. A. Vagin , and E. J. Dodson , Acta Crystallographica Section D, Structural Biology 53 (1997): 240–255. 10.1107/S0907444996012255 15299926

[8] J. Agirre , M. Atanasova , H. Bagdonas , et al., Acta Crystallographica Section D, Structural Biology 79 (2023): 449–461. 37219590 10.1107/S2059798323003510 PMC10233620

[9] R. P. Joosten , F. Long , G. N. Murshudov , and A. Perrakis , IUCrJ 1 (2014): 213–220. 10.1107/S2052252514009324 PMC4107921 25075342

[10] C. J. Williams , J. J. Headd , N. W. Moriarty , et al., Protein Science 27 (2018): 293–315. 29067766 10.1002/pro.3330 PMC5734394

[11] W. Kabsch and C. Sander , Biopolymers 22 (1983): 2577–2637. 6667333 10.1002/bip.360221211

[12] E. Krissinel , and K. Henrick , Journal of Molecular Biology 372 (2007): 774–797. 17681537 10.1016/j.jmb.2007.05.022

[13] S. M. Kuhn , M. Rubini , M. A. Müller , and A. Skerra , Journal of the American Chemical Society 133 (2011): 3708–3711. 21341705 10.1021/ja1099787

[14] J. D. Pédelacq , S. Cabantous , T. Tran , T. C. Terwilliger , and G. S. Waldo , Nature Biotechnology 24 (2006): 79–88. 10.1038/nbt1172 16369541

[15] S. Maniam , M. M. Cieslinski , S. F. Lincoln , et al., Organic Letters 10 (2008): 1885–1888. 18410119 10.1021/ol8002145

[16] C. Nie , C. Liu , S. Sun , and S. Wu , ChemPhotoChem 5 (2021): 893–901.

[17] D. Wang , W. Zhao , Q. Wei , C. Zhao , and Y. Zheng , ChemPhotoChem 2 (2018): 403–415.

[18] C. Brown , Acta Crystallographica 21 (1966): 146–152.

[19] H. M. Bandara and S. C. Burdette , Chemical Society Reviews 41 (2012): 1809–1825. 22008710 10.1039/c1cs15179g

[20] C. Brown , Acta Crystallographica 21 (1966): 153–158.

[21] A. Mostad and C. Rømming , Acta Chemica Scandinavica 25 (1971): 3561–3568. 10.3891/acta.chem.scand.25-3549 5151172

[22] E. Rusu , S. Shova , and G. Rusu , Acta Crystallographica Section E Structure Reports Online 67 (2011): o2333. 22065404 10.1107/S160053681103193X PMC3200752

[23] A. Reiter , A. Skerra , D. Trauner , and A. Schiefner , Biochemistry 52 (2013): 8972–8974. 24295282 10.1021/bi4014402

