# Supplementary material for: Structural Basis of the Light‐Switchable Interaction between an Azobenzene Side Chain in a Biosynthetic Protein and α‐Cyclodextrin
Source: ChemistryOpen. 2025 Nov 19;15(1):e202500471. doi: 10.1002/open.202500471 (PMC12877305; doi:10.1002/open.202500471)
Supplement: Supplementary file 1 — Supplementary Material [file OPEN-15-e202500471-s001.pdf]

Structural basis of the light-switchable interaction between an azobenzene side chain in a biosynthetic protein and α-cyclodextrin

Andreas Eichinger, Peter Mayrhofer, Markus R. Anneser, Leonie Jarzinka and Arne Skerra

Chair of Biological Chemistry, School of Life Sciences, Technical University of Munich, Emil-Erlenmeyer-Forum 5, 85354 Freising, Germany

Supporting Information

Table S1. X-ray data statistics.

	$sfGFP^{39Pap}\cdot\alpha$-CD
**Crystal Data:**	
Space group	$P4_3$ (#78)
Unit cell dimensions:<br>a, b, c [Å]<br>α, β, γ [°]	 69.27, 69.27, 115.35<br>90.0, 90.0, 90.0
Molecules per a.u.	2
**Data Collection:**	
Wavelength [Å]	0.97630
Resolution range [Å][a]	30.24–2.05 (2.15–2.05)
I/σ[I][a]	14.32 (5.51)
R_{meas} [%][a]	13.6 (82.2)
Unique reflections	32957
Multiplicity[a]	3.9 (3.9)
Completeness[a]	96.6 (99.2)
**Refinement:**	
R_{cryst} / R_{free}	18.8 / 22.8
Protein atoms	3583
Average B-factor [Å^2]	30.7
**Geometry:**	
R.m.s.d. bond lengths, angles [Å, °]	0.004, 1.212
Ramachandran analysis[b]:<br>core, allowed, generously allowed, disallowed [%]	 91.7, 8.3, 0.0, 0.0

[a] Values in parentheses are for the highest resolution shell.

[b] Calculated with PROCHECK

```
                       1         10         20         30
sfGFP(39Pap)     . S K G E E L F T G V V P I L V E L D G D V N G H K F S V R    30
wt-GFP           M S K G E E L F T G V V P I L V E L D G D V N G H K F S V S    30

                       40         50         60         70
G E G E G D A T X G K L T L K F I C T T G K L P V P W P T L V T T L T Y G V Q C    70
G E G E G D A T Y G K L T L K F I C T T G K L P V P W P T L V T T F S Y G V Q C    70

                       80         90         100        110
F S R Y P D H M K R H D F F K S A M P E G Y V Q E R T I S F K D D G T Y K T R A   110
F S R Y P D H M K Q H D F F K S A M P E G Y V Q E R T I F F K D D G N Y K T R A   110

                       120        130        140        150
E V K F E G D T L V N R I E L K G I D F K E D G N I L G H K L E Y N F N S H N V   150
E V K F E G D T L V N R I E L K G I D F K E D G N I L G H K L E Y N Y N S H N V   150

                       160        170        180        190
Y I T A D K Q K N G I K A N F K I R H N V E D G S V Q L A D H Y Q Q N T P I G D   190
Y I M A D K Q K N G I K V N F K I R H N I E D G S V Q L A D H Y Q Q N T P I G D   190

                       200        210        220        230
G P V L L P D N H Y L S T Q S V L S K D P N E K R D H M V L L E F V T A A G I T   230
G P V L L P D N H Y L S T Q S A L S K D P N E K R D H M V L L E F V T A A G I T   230

H G M D E L Y Q S A W S H P Q F E K   248
H G M D E L Y K . . . . . . . . . .   238
```

Figure S1. Amino acid sequence of sfGFP[39Pap] aligned against the one of wild-type GFP from *Aequorea victoria* (UniProt-ID: P42212) and with secondary structure assignments according to the present X-ray structural analysis. Differing residues are highlighted and the Pap residue (X) at position 39 is indicated by a star.

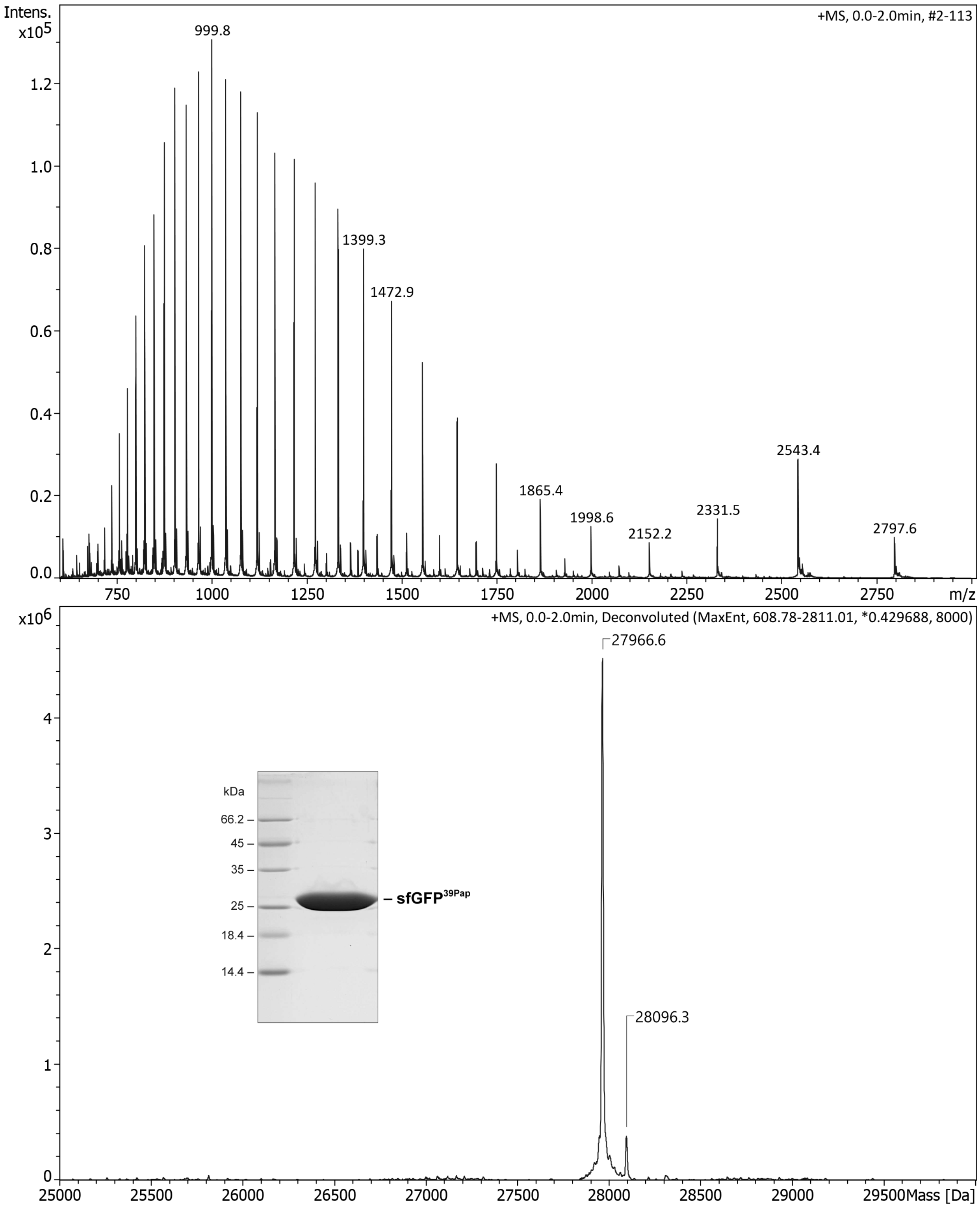

Figure S2. ESI mass spectrum of the purified sfGFP^{39Pap} that was used for cocrystallization with α-CD. Inset: Coomassie-stained SDS-PAGE of the purified sfGFP^{39Pap}. Calculated mass for the protein with processed N-terminal Met residue and mature fluorophore: 27,966.45 Da. The small peak at higher mass corresponds to a minor contamination by the recombinant protein that has the start Met residue retained (calc.: 28,097.57 Da).

```
from chempy import cpv
import numpy, math
def plane_normal(selection):
   stored.x = list()
   cmd.iterate_state(-1, selection, 'stored.x.append([x,y,z])')
   x = numpy.array(stored.x)
   U,s,Vh = numpy.linalg.svd(x - x.mean(0))
   return cpv.normalize(Vh[2])
dir1 = plane_normal('/9S0T//A/OZW`39/CG+CD1+CD2+CE1+CE2+CZ')
dir2 = plane_normal('/9S0T//A/OZW`39/C6+C2+C1+C4+C3+C5')
print('Angle in degrees:', math.degrees(cpv.get_angle(dir1, dir2)))
```

Figure S3. Python script "AromaticAngle.py" to calculate the angle (α) between the two phenyl ring planes in Pap (or other azobenzene derivatives) to be executed using PyMOL ver. 3.1.6.1 software (adapted from https://pymol-users.narkive.com/O0BZdPOe/pymol-how-to-measure-the-angle-between-two-aromatic-rings).
